# Lithuanian midwives’ attitudes and actions during low-risk birth

**DOI:** 10.18332/ejm/166294

**Published:** 2023-06-28

**Authors:** Alina Liepinaitienė, Ema Cilinskaitė, Aurimas Galkontas, Audrius Dėdelė

**Affiliations:** 1 Department of Environmental Sciences, Faculty of Natural Sciences, Vytautas Magnus University, Kaunas, Lithuania; 2 Faculty of Medicine, Kauno Kolegija Higher Education Institution, Kaunas, Lithuania; 3 Public Institution Kaunas City Polyclinic, Kaunas, Lithuania; 4 Faculty of Public Health, Medical Academy, Lithuania University of Health Sciences, Kaunas, Lithuania

**Keywords:** midwives’ attitude, midwives’ actions, low-risk birth, Lithuanian midwives, attitude during low-risk birth, actions during low-risk birth

## Abstract

**INTRODUCTION:**

This study sheds light on the attitudes and practices of Lithuanian midwives during low-risk births. The aim is to reveal how autonomous work is incorporated into daily routines, how care is oriented towards the mother, and how care is delivered before and during interventions. It highlights midwives’ views on both their own and their colleagues’ actions during labor, what is aimed for and what outcome is expected.

**METHODS:**

A qualitative research method was chosen. Midwives were interviewed individually in February and April 2022 by random sampling and semi-structured interviews, after the purpose of the survey was explained and their consent was given to use the information only for scientific work purposes. Midwives were recruited through social networks, sharing information about the study and its nature. All data were coded and analyzed in aggregate form. Ten midwives working in the labor ward participated in the study.

**RESULTS:**

From the midwives’ point of view, every birth and its experience are unique. Midwives work together with mothers to achieve the common goal of a positive birth experience. Communication with the mother and her family, good rapport, clear information and informed decision-making are key aspects for midwives during labor. The midwife’s actions must be reasonable and purposeful, with a preference for non-medicated methods of pain and stress relief.

**CONCLUSIONS:**

A low-risk birth that is within the competence of midwives is one in which there is a low likelihood of medical interventions. Midwives are encouraged to minimize the use of interventions and to provide high quality delivery care.

## INTRODUCTION

Childbirth is a life-changing experience that creates lifelong memories, so midwives need to be aware of the different needs of women in labor, emotional, physical, and informational. A good relationship can not only reduce the fear associated with childbirth, but also contribute to a positive birth experience later on. Continuous support during labor reduces the need for medical interventions and can improve the well-being of both mother and newborn^1^. Over the last two decades, there has been a dramatic increase in the number of interventions^2,3^ aimed at encouraging, accelerating, monitoring the normal, physiological process of labor. This has led to a debate about what is ‘low-risk’ in childbirth, but this concept has not been standardized. A medicalized birth process can be harmful to the mother and provide a negative experience^2^. This is why the midwife’s attitude to work is very important, a safe environment provides an excellent, stable basis for creating a positive birth experience, and patience is considered a necessity^3^. As interventions and technologies can make the midwife less attentive to the mother, without constant support and presence, and can make the woman feel neglected, confused, and lonely. Caring and good communication skills bring the woman closer to the midwife, making her feel safe and listened to^1^. To understand the problem of the theme, they were presented with the aim of the study, which is about revealing the attitudes and activities of midwives in low-risk labor.

## METHODS

### Study design and collection of data

A literature review was conducted to explore midwives’ views on physiological childbirth and its use in practice. Open access reviews were used from all around the world, based on publications over the last 10 years. A qualitative research strategy was chosen for the empirical study in order to gain a broader understanding of midwives’ views. Data were collected using a semi-structured interview method and data analysis was conducted using qualitative content analysis. Participants were interviewed through remote meetings, answers to questions were recorded on a dictaphone with the consent of the participants, an explanation was given of the purpose of the interview and their consent obtained to use the information provided for research purposes only. The study maintained privacy, confidentiality, anonymity, and the midwives participated voluntarily and were free to discontinue participation at any time.

The study used semi-structured interviews, with the same basic requests for all participants: 1) Explain how you understand midwives’ attitudes during low-risk birth, and 2) Explain how you understand midwives’ actions during lowrisk birth. The interview requests were structured, but at the same time flexible enough for them to share personal work experiences.

The participants were selected by non-probability purposive sampling. On average, the interviews lasted 83 minutes (the shortest was 49 minutes and the longest was 214 minutes). The length of the interviews depended on the eloquence of the participants and their willingness to share their experiences, and the timing of the interviews was coordinated with each participant personally in order to avoid rushing and extraneous distractions. Those who agreed to take part in the study were cordial and willing to share their experiences and views.

### Participants

Midwives were recruited through social media, sharing information about the study and its nature, and inviting people to join the study. All data were coded and analyzed in aggregate form. The study took place between February and April 2022. Participants were selected according to the following criteria: able to speak Lithuanian; work in the delivery department as a midwife; and agree to participate in the study.

Ten midwives working in the maternity unit participated. The majority of midwives who participated in the study were midwives who had been working in midwifery for more than 10 years, and midwives were also older than 30 years. The study involved 5 participants each from level III (tertiary level facilities providing specialized obstetric and neonatal care) and level II B (secondary level B facilities providing skilled obstetric and neonatal care) hospitals where midwives attend low-risk births independently (Table 1). The collected interview data were analyzed in a summarized manner using qualitative content analysis. Interview voice recordings and researchers’ notes were transcribed immediately after the interviews and read multiple times during the analysis, highlighting the main themes and sub-themes, and making interpretations of them. The results obtained are supported by the literature analysis and scientific sources.

**Table 1:** Sociodemographic data of midwives who participated in the research between February and April 2022 (N=10)

*Midwife No.*	*Age (years)*	*Years of service in midwifery*	*Degree*	*Maternity hospital level*
1	26	4	Higher university	III
2	35	13	Higher non-university	III
3	35	9	Higher university	II B
4	27	4	Higher non-university	II B
5	26	3	Higher non-university	II B
6	48	26	Higher university	II B
7	39	10	Higher university	III
8	44	20	Higher university	II B
9	59	23	Higher university	III
10	64	40	Higher non-university	III

## RESULTS

Results on midwives’ attitudes and activities in low-risk births are presented. In order to uncover midwives’ attitudes and personal experiences, the interview data were analyzed into the themes: ‘Midwives’ attitudes’, and ‘Midwives’ actions’ (Table 2).

**Table 2:** Main themes with sub-themes about midwives’ attitudes and actions during low-risk birth, obtained from interviews (N=10)

*Themes*	*Sub-themes*
**Attitudes**	Towards low-risk childbirth
	Towards interventions
	Towards non-clinical care of childbirth
**Actions**	During low-risk childbirth
	Use of interventions
	Non-clinical care of childbirth

### Midwives’ attitudes

In Lithuania, midwives are able to independently care for the low-risk mother, to accept spontaneous labor and to independently administer non-medicated pain relief during labor.

*Midwives’ attitudes towards low-risk childbirth*

The attitude of the midwife attending the birth is important for the best possible care:

*‘Every birth is unique in its experience.’* (Midwife No. 3)

Exclusively for midwives of low-risk deliveries, only midwife-led care should be encouraged. The study participants agreed that childbirth:

*‘…is about the mother and midwife working together for the best outcome.’* (Midwife No. 2)

and

*‘... the goal of childbirth is a healthy newborn and mother.’* (Midwife No. 5)

The bond between the midwife and the mother is important during childbirth, and midwives need to be committed to their work and to promote low-risk births and encourage women to give birth exclusively under the care of the midwife.

*Midwives’ attitudes towards interventions*

The attitudes of the midwives in the study towards low-risk interventions divided into two groups. All participants emphasized that the condition of both the mother and the fetus/newborn should be good. Some of them stated that:

*‘In a low-risk birth, the mother does not need additional interventions. The whole period of labor develops on its own, with the help of a relative, and non-medicated anesthesia.’* (Midwife No. 8)

*‘Childbirth without any medical interventions ... The woman arrives at the maternity home, after the natural labor has started, gives birth without episiotomy [even with a small tear ...], the placenta separates itself, a healthy newborn is born and the mother is transferred to the postnatal ward after 2 hours.’* (Midwife No. 6)

Others disagreed, stating that childbirth is low-risk even after epidural anesthesia, amniotomy, and episiotomy:

*‘In my opinion, I think that childbirth is low-risk when the mother is healthy and the child is healthy, regardless of whether there was an epidural, whether there was an episiotomy, whether there was an amniotomy.’* (Midwife No. 1)

*‘Amniotomy could be considered part of a low-risk birth when it is performed after labor has started. I could also classify episiotomy as part of a low-risk delivery.’* (Midwife No. 5)

*‘I would include amniotomy as one of the interventions of natural childbirth. It is a minimal intervention to help the mother to get the labor started faster.’* (Midwife No. 7)

*Midwives’ attitudes towards non-clinical care of childbirth*

The relationship that is created between the midwife and the woman is important:

*‘I try to involve the mother as much as possible in the whole process of labor, to tell her everything that is happening, what is going to happen. Then they become calmer.’* (Midwife No. 4)

*‘It is very important to me that all my patients feel safe to give birth according to the plan they want.’* (Midwife No. 3)

*‘Throughout the birth, every effort is made to communicate with the patient and her family and to pass on all useful information ... The mother is fully involved in the process.’* (Midwife No. 9)

The phenomenon of ‘being with the woman’ is at the heart of midwifery and underpins the philosophy, practice and relationships of midwifery. This relationship extends not only to the woman and the midwife, but also to the relatives who support the woman. Being ‘with the woman’ and in the context of the relationship during childbirth, facilitates woman-centered care. Being ‘with the woman’ has an impact on midwives and especially on the mothers with whom they work.

### Midwives’ actions

Midwives’ professional autonomy and the ability to build meaningful relationships are the most frequently mentioned positive aspects.

*Midwives’ actions during low-risk childbirth*

Midwife-led care is beneficial and has no negative consequences for both the mother and the newborn:

*‘I worked for a year in the maternity ward, attending and receiving low-risk births on my own, and medium-risk and high-risk births under the supervision of a doctor.’* (Midwife No. 1)

*‘I always try to take into account what my patient is like. If she wants to move - I provide opportunities to move. If she doesn’t want to move, but just lie in bed, I don’t get up and make her move. But I always try to explain what is better, what can be achieved one way or the other, and if the patient agrees and tries it, it is up to her whether or not she wants to continue to do as the midwife recommends.’* (Midwife No. 10)

Strategies for antenatal care can be divided into four main groups: support for women during labor, relaxation and pain relief, care during labor with minimal intervention and preparation for labor.

*Midwives’ actions in the use of interventions*

Respondents agree that the aim of low-risk childbirth is to avoid unnecessary medical interventions, to allow the woman to give birth as naturally as possible, and to allow her to feel the birth fully:

*‘Most of my mothers are low-risk, so I always try to offer as few interventions as possible during labor. Women need to experience the benefits and ‘pleasure’ of natural childbirth.’* (Midwife No. 7)

They also agreed that only non-medicated methods should be used to relieve pain:

*‘Only natural methods of pain relief: movement, massage, exercise, abdominal caresses, etc.’* (Midwife No. 3)

Some respondents mention cardiotocography:

*‘There are no medical interventions that can be attributed to low-risk childbirth. The only procedure, a test that could be classified as an operational procedure for labor is the writing of a cardiotocography during labor, but only when necessary to assess the condition of the fetus.’* (Midwife No. 9)

*Midwives’ actions in non-clinical care of childbirth*

Regarding non-clinical delivery care, respondents were unanimous in their opinion: hydrotherapy (shower, bath), a gymnastic ball, massage to relieve stress and pain, and the importance of finding a position that suits the mother herself:

*‘Shower for pain relief, back massage, finding the right position, bath.’* (Midwife No. 2)

*‘I usually recommend women to take a shower. I notice that women like it and like it. The water is relaxing, it relieves pain.’* (Midwife No. 1)

Music, dance, breathing exercises, thought diversion were also mentioned:

*‘... we also suggest to the patients to listen to music, try to read a book or do a favorite activity. It’s great fun when opera soloists or singers use breathing exercises while singing.’* (Midwife No. 9)

*‘... dancing with a partner, relaxing music.’* (Midwife No. 4)

Another very important element in creating a positive birth experience is the environment, the people around you, the provision of information:

*‘... providing a calm and comfortable environment in the labor ward, communication, support, encouragement, following recommendations, non-medicated methods of anesthesia, sharing decision-making with the patient, involving the partner in the labor.’* (Midwife No. 5)

However, mothers need to remember that it is not only the midwife or other staff who are responsible for the birth, but also the mother herself:

*‘It depends very much on the situation, but a lot depends on the woman’s concentration at the most important moments and her ability to stay focused, to collaborate, to listen and to follow commands ...’* (Midwife No. 5)

## DISCUSSION

In many countries around the world, midwives are the main providers of women’s healthcare during pregnancy and childbirth. With the continuous improvement of medical care, medical interventions act as an additional threat if they are used without any clinical indication and without any ethical considerations^4^. The attitude of the midwife attending the birth is important for the best possible care. Exclusively midwife-led care for low-risk mothers reduces not only the likelihood of cesarean section surgery, but also the number of other medical interventions, without putting the newborn at risk. For low-risk deliveries, only midwife-led care should be encouraged^5^. Midwives attending low-risk births are the key persons who can provide respectful maternity care and support women in making informed decisions about childbirth. Different midwives may not have the same views, influenced by context, prior beliefs, and values^6^. Communication with the mother and her companions, involvement in the birth process, and support for informed decision-making are crucial aspects of a positive birth experience^7^. The care provided must meet the needs of midwives and birthing mothers, and must be created by midwives themselves in the course of their work^8^. For a positive birth experience, well-chosen care is essential and midwife-led care should be provided for all low-risk births^9^. Early assessment of labor to minimize obstetric interventions and preparation for labor according to an individual birth plan, presence of a trained birth partner, relaxation with massage and music to avoid a negative birth experience have been identified^10^. In low-risk labor, continuous cardiotocography should not be performed and intermittent fetal cardiac auscultation is preferred^11^. In the United States, certified nurse midwives are routinely recognized as independent advanced practice nurses who can provide prenatal care. Midwifery-led care is considered high-touch/low-intervention and is based on a philosophy of care in which pregnancy and childbirth are normal life events for many women^12^.

Midwife-led care, even in a very complex tertiary hospital system, has a significant positive impact on women’s outcomes. In particular, among women whose deliveries were supervised exclusively by a midwife, there was a higher rate of spontaneous deliveries by natural routes and fewer medical interventions, a reduction in the use of oxytocin to speed up the delivery process, a reduction in the use of painkillers and a reduction in the incidence of amniotomies and episiotomies. Nevertheless, no adverse effects on maternal and neonatal outcomes were observed^9^.

In Lithuania, when a woman is admitted to a maternity hospital and is diagnosed in labor, after an initial examination, the risk during labor is assessed and the mother is assigned to a risk level. Although the risk may vary throughout labor, correct risk assessment is crucial for the subsequent management of labor.

Low-risk births can be self-managed by a midwife^13^. The social situation of midwifery in some countries (e.g. middle-income countries) increasingly threatens to reduce the effectiveness, impact or capacity of midwives themselves. Technological advances and medical interventions act as an additional threat when they are used without any clinical indication and in complete disregard of ethical considerations. Maternity care and routine delivery tactics vary around the world. Greater attention needs to be paid to the education of midwives and women at a global level, with particular emphasis on ethics, communication and the philosophy of care itself^4^. Pain relief methods have controversial effects, and of all the methods of labor relaxation, only massage and music are recommended to improve a woman’s birth experience^10^.

From the point of view of the interviewed midwives in Lithuania, the most important aspect of non-clinical childbirth care is communication with the mother and her relatives, creating a good mutual relationship and providing clear, easy-to-understand information. Comparing early amniotomy with late amniotomy, or spontaneous rupture of the fetal membranes, in the presence of a mature cervix, amniotomy did not increase the risk of cesarean section, but also reduced the overall time to labor^14^.

The routine use of various interventions such as episiotomy, electronic fetal monitoring and pain control has increased dramatically in recent years, although they have not been shown to improve maternal or neonatal outcomes^9^. Factors associated with the increased medicalization of childbirth contribute to the midwife’s responsibility for low-risk women and births. Midwives need to take responsibility for bringing about change, as their professional identity is at stake^15^.

### Strengths and limitations

This is the first study of its kind in Lithuania to explore midwives’ attitudes towards low-risk births. However, the limitations of the study are due to different perceptions of what constitutes a low-risk birth. It is also due to the low involvement of midwives in the study because findings were similar with low variability in answers.

## CONCLUSIONS

Low-risk birth is a birth with a low likelihood of medical interventions, the care provided is within the midwife’s professional competence, the birth is spontaneous, the mother is classified as low-risk at the start of the birth and remains so throughout the birth. Midwives have a unique experience of childbirth, and they work together with their mothers to achieve the common goal of a positive birth experience. There was unanimity of views on non-clinical antenatal care, with communication with the mother and her relatives, good rapport and provision of information being the most important aspects in low-risk labor. During low-risk labor the midwife’s actions should be reasonable and purposeful in order to provide the best possible birth experience. Midwives prefer non-medicated pain and stress relief and informed decision-making.

## Data Availability

The data supporting this research are available from the authors on reasonable request.
